# Cutis verticis gyrata treated with intralesional hyaluronidase

**DOI:** 10.1016/j.jdcr.2025.06.021

**Published:** 2025-06-24

**Authors:** Milan Hirpara, Adaora Ewulu, Natasha A. Mesinkovska

**Affiliations:** Department of Dermatology, University of California Irvine, Irvine, California

**Keywords:** cutis verticis gyrata, hyaluronidase, paquidermia verticis gyrata, scalp injection

## Introduction

Cutis verticis gyrata (CVG) is a rare scalp disorder presenting as prominent furrows and ridges resembling the cerebral cortex’s gyri. CVG is predominantly seen in males, with a reported prevalence of approximately 1 in 100,000 males.^1^ CVG can be classified as either primary, which is idiopathic, or secondary, which results from underlying conditions, such as acromegaly, Turner syndrome, and anabolic steroid use. Primary CVG includes 2 subtypes: essential, which occurs in isolation, and nonessential, which is associated with intellectual, neuropsychiatric, or ophthalmologic abnormalities.^1^ When secondary CVG is suspected, an endocrinologic evaluation is recommended to rule out underlying systemic disease. Therapeutic modalities for CVG are surgical and include scalp reduction surgery with expanded skin flaps.^1^ Herein, we present a case of CVG secondary to anabolic steroid use managed with intralesional hyaluronidase as a nonsurgical therapeutic approach.

## Case report

A 70-year-old male, a former bodybuilder, presented with a 30-year history of thickened scalp skin with grooves. His medical history includes androgenetic alopecia status post 2 hair transplant procedures performed 12 and 10 years ago. He denied any associated symptoms such as itching, burning, and bleeding. The condition developed gradually, without trauma, seizures, or focal neurological deficits. Prior to presentation, a hormonal workup was completed by an outside facility, revealing normal thyroid function and insulin-like growth factor 1 levels.

The patient reported an 8-year history of anabolic steroid use during his twenties, when he competed in bodybuilding. He primarily used oral oxandrolone (10 mg daily) and intramuscular nandrolone (1 mL of 100 mg/mL biweekly). At 40 years old, a decade after discontinuing anabolic steroids, he started using topical testosterone cream (100 mg daily) applied to his forearms. Within a year of initiating this regimen, his hair loss worsened, and he coincidentally noticed multiple folds of skin over his scalp.

**Fig 1:**
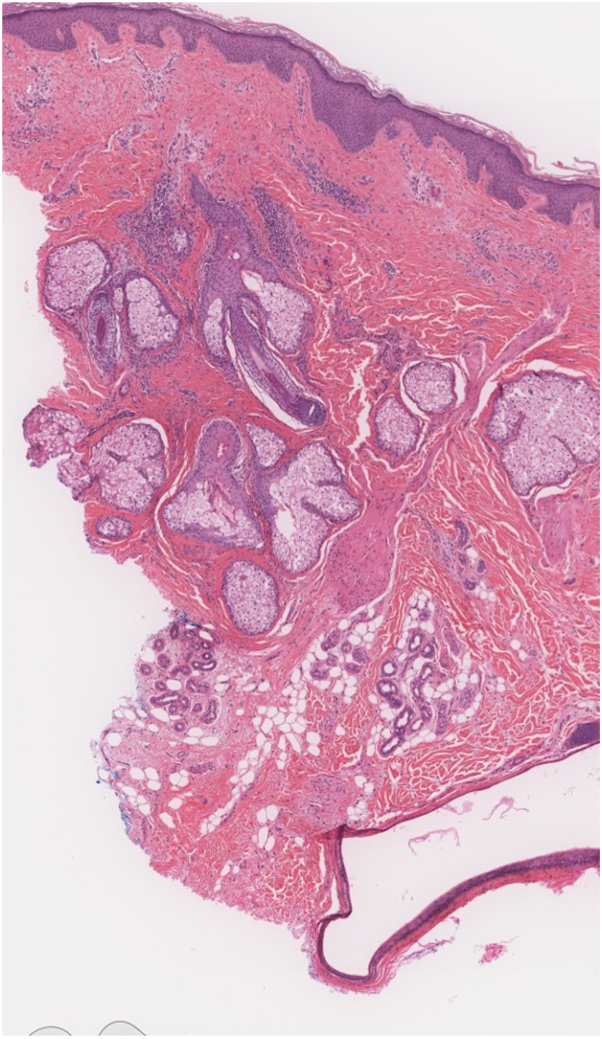
Histopathologic findings of scalp gyri in cutis verticis gyrata. Histology shows mild fibrosis of the perifollicular adventitial dermis, mild perivascular and perifollicular lymphocytic infiltrate with occasional histiocytes concentrated around the hair bulge area, and focal follicular dropout with increased dermal fibrosis. Additionally, there was a large epidermal inclusion cyst lined by squamous epithelium.

Physical examination revealed a young-for-age male with a muscular habitus and findings of deep, indurated gyrate skin folds extending from the frontal to occipital scalp. The elevated gyri were most pronounced around the frontotemporal angles bilaterally. He also had marked seborrhea, folliculitis, and decreased hair density along the crown and frontal scalp. Clinical diagnosis of CVG was established, and a biopsy of the right vertex was performed. Histopathological examination showed superficial and deep perivascular and perifollicular lymphocytic infiltrate, significantly increased dermal fibrosis, and epidermal inclusion cysts (Fig 1). The diagnosis of CVG was suspected to be secondary to anabolic steroid use.

**Fig 2:**
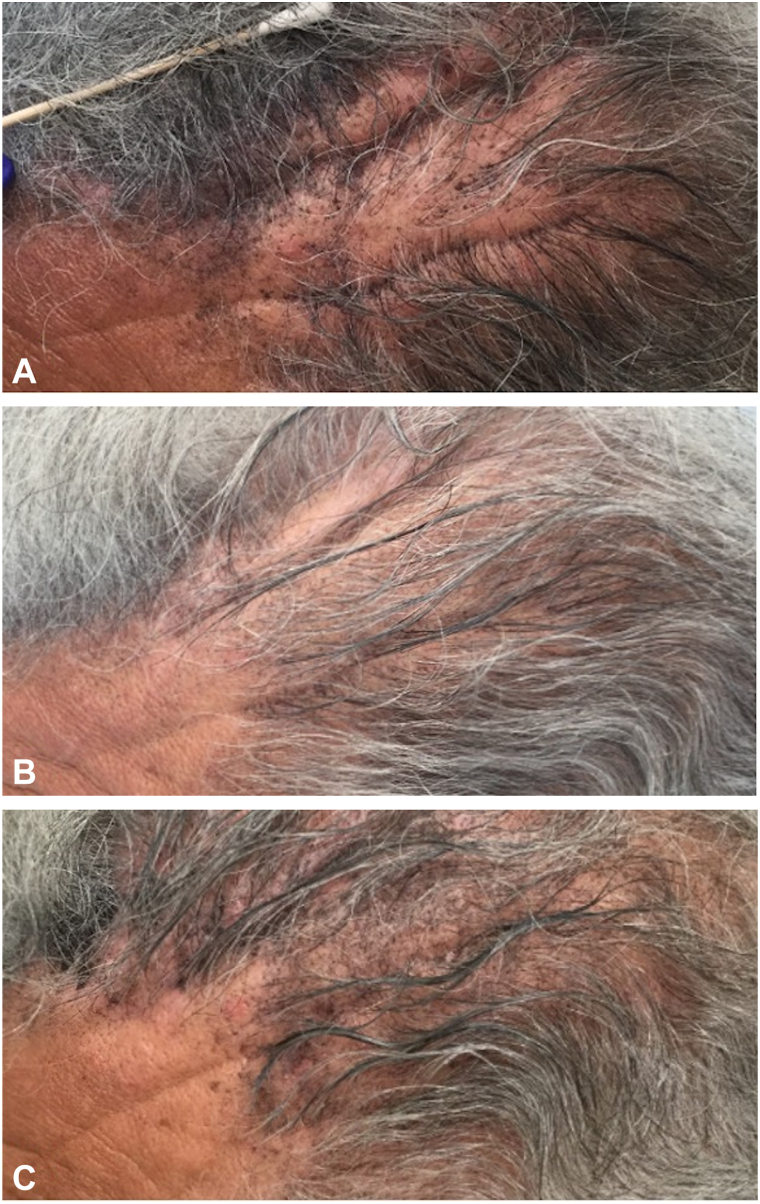
Visual improvement of cutis verticis gyrata following intralesional hyaluronidase (4 treatments at 6-week intervals). **A,** After 3 weeks (150 U hyaluronidase). **B,** After 6 weeks (300 U hyaluronidase). **C,** After 13 weeks (450 U hyaluronidase).

Initial management targeted the biopsy-confirmed epidermal cyst with serial intralesional triamcinolone (2 mL of 5 mg/mL and 2 mL of 10 mg/mL) every 8 to 12 weeks over 2 years. To relax the scalp musculature, he also received 2 courses of onabotulinumtoxinA (20 U and 50 U) with some improvement; however, the patient was subsequently lost to follow-up for 3 years. After observing progression of his condition, the patient returned for further treatment. He was treated with intralesional hyaluronidase (150 U/mL, 4 to 8 weeks), which yielded significant softening of the tissue and visible flattening of the furrows (Fig 2). Moderate reduction in comedones and increased hair density were also noted in treated areas, though the most significant improvement was in the scalp folds. The patient tolerated the regimen well and was satisfied, with no adverse effects, and continues to receive hyaluronidase injections bimonthly with sustained improvement in scalp texture and appearance.

## Discussion

We demonstrate the effectiveness of intralesional hyaluronidase injections as a cost-effective, minimally invasive, and well-tolerated alternative for the management of CVG.

CVG has a strong endocrinological component supported by its onset after puberty, reported regression following castration, and association with endocrine disorders such as acromegaly and Klinefelter syndrome.^1^^,^^2^ CVG patients have also been found to have significantly lower testosterone levels potentially linked to exogenous testosterone use.^2^ Anabolic steroids and testosterone have been shown to stimulate sebaceous gland hypertrophy, upregulate collagen production, and increase skin thickness.^3^, ^4^, ^5^ Although a prior case described CVG developing after months of continuous exposure to 15 different anabolic steroids, our case demonstrates a delayed onset after initiating topical testosterone, nearly a decade after an 8-year history of using just 2 anabolic agents.^5^ This suggests that cumulative androgen exposure, even years apart, may contribute to CVG pathogenesis.

Hyaluronidase degrades hyaluronic acid (HA), a key component of the dermal extracellular matrix. HA contributes to structural integrity, dermal volume, and tissue hydration. Several case reports on CVG have identified increased dermal thickness, with 1 demonstrating greater HA deposits compared to control specimens.^6^^,^^7^ Histopathology in CVG can range from normal skin architecture to accumulation of HA in the dermis. Hyaluronidase enzymatically cleaves HA into its 2 constituent monosaccharides, consequently loosening the skin and reducing scalp rigidity.^8^ Hyaluronidase may also improve local tissue diffusion and lymphatic drainage, further softening the folds.

While hyaluronidase is generally well-tolerated, common adverse effects include localized injection site reactions, headache, and fatigue.^8^ Rarely, serious complications such as hypersensitivity reactions, anaphylaxis, and thromboembolism may occur.^8^ The risk of anaphylaxis is of particular concern due to the possibility of prior sensitization, especially in individuals with a history of allergic reactions or exposure to animal or insect stings, which naturally contain hyaluronidase.^8^ Allergy skin testing and proper hydration before administering hyaluronidase can mitigate the potential for hypersensitivity reactions and thromboembolism, respectively.^8^ In our case, sensitivity testing was not performed, considering the patient had no history of allergies or anaphylaxis. However, he was monitored closely during and after each injection.

Treatment options for CVG remain limited, particularly for patients seeking cost-effective, noninvasive solutions. Surgical management, including scalp excision, flap reconstruction, and tissue expansion, can be invasive and costly. In our case, hyaluronidase was prescribed through the patient’s pharmacy at approximately $11 per vial and administered in clinic, offering a practical and affordable alternative. The observed improvement highlights the potential of hyaluronidase as a therapeutic option and aligns with 2 prior case reports, which used similar regimens—150 U/mL every 6 weeks for 6 treatments and 200 U/mL every 6 weeks for 3 treatments.^9^^,^^10^ Prospective studies are necessary to establish the optimal treatment protocols for long-term efficacy.

## Conflicts of interest

None disclosed.
